# Phrase Depicting Immoral Behavior Dilates Its Subjective Time Judgment

**DOI:** 10.3389/fpsyg.2021.784752

**Published:** 2021-12-24

**Authors:** Lina Jia, Bingjie Shao, Xiaocheng Wang, Zhuanghua Shi

**Affiliations:** ^1^Department of Education, School of Education, Jiangnan University, Wuxi, China; ^2^Department of Psychology, Ludwig-Maximilians-Universität München, Munich, Germany

**Keywords:** time perception, emotion, immoral phrases, disgust phrases, embodied timing

## Abstract

Intuitive moral emotions play a major role in forming our opinions and moral decisions. However, it is not yet known how we perceive the subjective time of moral-related information. In this study, we compared subjective durations of phrases depicting immoral, disgust, or neutral behaviors in a duration bisection task and found that phrases depicting immoral behavior were perceived as lasting longer than the neutral and disgusting phrases. By contrast, the subjective duration of the disgusting phrase, unlike the immoral phrase, was comparable to the neutral phrase. Moreover, the lengthening effect of the immoral phrase relative to the neutral phrase was significantly correlated to the anonymously prosocial tendency of the observer. Our findings suggest that immoral phrases induce embodied moral reaction, which alters emotional state and subsequently lengthens subjective time.

## Introduction

When reading newspaper headlines or browsing internet news, many of us are often captured by moral-related news as compared to other politics-related news, and our emotions follow the story. Such attentional capture and intuitive reaction seem to be very natural for us. Indeed, studies have shown that moral-related stimuli are often prioritized over nonmoral stimuli (Gantman and Van Bavel, 2014; Gantman et al., 2020). For example, moral words were easier discriminated than nonmoral words when they were presented very shortly (Gantman and Van Bavel, 2014; Gantman et al., 2020). Reading news about immoral behavior also causes us a negative emotion. However, it is not yet known if such negative emotion induced by an immoral stimulus would also lengthen its subjective time, given that it remains controversial regarding subjective time distorted by negative emotion (Droit-Volet et al., 2013; Lake, 2016; Droit-Volet, 2019). Some studies using high-arousing negative emotion have shown lengthening effect of subjective time (Angrilli et al., 1997; Noulhiane et al., 2007; Fayolle et al., 2015; Droit-Volet and Berthon, 2017), while others have argued that different types of negative emotions link to their distinct behavioral functions, which may yield differential subjective distortions (Gil and Droit-Volet, 2011; Shi et al., 2012; Grondin et al., 2014). Even worse, the connection to the subjective time of stimuli related to morality has not been formally investigated. On this ground, the aim of this study was therefore to investigate the relationship between morality and time perception. In the following, we first briefly review relations between time perception and emotion, and its connection to embodiment and morality. Then we hypothesize the relations between moral information and subjective time, and in the end, we propose the experimental design to verify our hypotheses.

### Emotion and Time Perception

Emotion is often evoked and coupled by different types of moral behaviors (Tangney et al., 2007), while emotion can subsequently influence time judgment. Most studies investigating mechanisms underlying emotional time perception have applied simple affective stimuli (Droit-Volet and Gil, 2009; Droit-Volet et al., 2013) such as emotional images selected from International Affective Pictures System (Angrilli et al., 1997), emotional facial expressions (Effron et al., 2006), looming/receding movement stimuli (van Wassenhove et al., 2011; Jia et al., 2015), emotional clips of the film (Fayolle et al., 2014), and effective sounds (Noulhiane et al., 2007). Studies using explicit manipulation of arousal levels and saliency (Angrilli et al., 1997; Gil and Droit-Volet, 2012; Fayolle et al., 2015) often reveal that the high level of an arousing affective stimulus lengthens its subjective time. Many findings (Droit-Volet et al., 2013; Lake, 2016; Droit-Volet, 2019) have been interpreted through the classical framework of pacemaker-accumulator clock, respectively (Gibbon et al., 1984; Zakay and Block, 1996), which assumes three essential components in time processing: a pacemaker, a switch, and an accumulator. A pacemaker generates internal pulses, which pass to the accumulator *via* a switch control. The switch turns on and off following the on and off of the to-be-timed event. Affective stimuli are often assumed to elevate internal arousal states (Droit-Volet et al., 2013; Fayolle et al., 2015), which subsequently increases the rate of a pacemaker, generating more pulses per unit of time to the accumulator. In addition, high-arousal affective stimuli capture attention, and more temporal pulses were passed *via* the switch, resulting in duration lengthening (Tse et al., 2004; Lui et al., 2011; Droit-Volet et al., 2013).

It should be noted that arousal is not the main determinant factor in the subjective percept of affective stimuli, the meaning of social interaction could also impact time perception. For example, multiple studies have shown emotional facial expressions, as the social-interaction emotion, can differentially distort the subjective duration (Effron et al., 2006; Gil and Droit-Volet, 2011; Grondin et al., 2014, 2015). For instance, Gil and Droit-Volet (2011) compared subjective durations of six emotional facial expressions (anger, fear, sad, happy, shame, and disgust), and found that durations of the faces expressing anger, fear, sad, and happiness were judged longer relative to the neutral face. Furthermore, the subjective duration of anger and fear expressions were estimated longer than the sad and happy ones, even when their arousal levels were comparable. By contrast, the duration of a shame face was often underestimated while a disgusted face did not induce any duration distortion. The authors interpreted their findings of time distortion according to the urgency of readiness to act on a receiving stimulus: people tend to fight or flee when they see anger or a fearful face of another person, while they approach toward a happy or sad person. These different reactions lead to overestimation of anger and fear relative to the happy or sad expressions. The sight of a disgusted face, however, cannot motivate people to react, thus the subjective time is not modulated by its disgust emotion. Shame faces, on the other hand, elicit a feeling of shame, mirroring back to their own internal state, such that less attention is shared to the temporal processing, which subsequently causes its duration underestimated (Droit-Volet et al., 2013).

### Embodiment, Morality, and Time Perception

There are several other recent studies that have also confirmed that implicit reaction caused by embodied affective states plays an important role in the perceived time (Droit-Volet et al., 2013; Grondin et al., 2014; Jia et al., 2015; Droit-Volet and Dambrun, 2019). For example, Jia et al. (2015) have shown that the subjective duration of a given tactile stimulus depends on whether participants can react to an external concurrent event (a moving ball) or not. When the ball is moving in the direction that participants can interact with, subjective time is lengthened as compared to the condition that the ball moving is irrelevant for reaction. In another recent study (Droit-Volet et al., 2020), participants saw an arm of a mannequin through virtual reality glasses. To produce out-body illusion, they were firstly stroked synchronous and asynchronous with the strokes to the mannequin and then were asked to judge the temporal interval between two touches to the body of the mannequin. Results showed that the subjective duration is perceived longer in the synchronous-stroking condition as compared to the asynchronous-stroking condition.

To be a social norm, individuals keep alert to their moral behaviors and react to moral events (Haidt, 2008; Haidt and Graham, 2009). Many of our moral judgments are closely linked to our embodied affective states (Haidt, 2001). For example, experimentally manipulated the heartrate of the observer seems to influence their moral judgments, with perceived faster heart rate leading to feelings of higher moral distress (Gu et al., 2013). Doing or seeing moral behaviors, on the other hand, can also influence affective states and perception. Behavioral and electrophysiologic studies have revealed that morality could enhance perception and awareness (Gantman and Van Bavel, 2014; Gantman et al., 2020). For instance, Gantman et al. (2020) recently reported that moral words are prioritized over nonmoral words in perceptual processing. Similarly, Anderson et al. (2011) have shown that enhanced perception for the neutral faces was previously paired with the description of negative social behavior. Notably, however, these studies mainly adopted target detection, identification, and lexical decision tasks. None of them has focused on time perception. Although the literature has shown the interplay of moral judgments and embodied affective states, little is known how the perception of a moral event, particularly with those immoral events, alters time judgment of the event.

On this ground, this study aimed to investigate whether time perception of moral events is coupled with moral perception. Given that perceiving immoral information often induces negative emotion and negative emotions differentially impact subjective time (e.g., the subjective time of the disgust emotion we reviewed above), we compared the subjective time of immoral information to both the neutral and negative disgust information. In addition, it should be noted that morality is an individual preference (Haidt, 2007; Saucier, 2018). Individual differences in moral preferences must be considered as an important factor mediating decision making and perception (Palmer et al., 2013; Yang et al., 2017). For example, Yang et al. (2017) investigated whether the individual difference in moral reference (measured by the Moral Foundation Questionnaire) influences the processing morality in a Rapid Stream Stimulation paradigm (Rudell, 1991). In their study, a recognizable word (depicting immoral behavior, disgust behavior, neutral behavior, or city/country name) was presented in a stream of nonrecognizable background stimuli. Each stimulus was shown for 250 ms without interstimulus intervals. Participants are usually required to respond as soon as they detect the name of a country or a city. Their results showed that the high-sensitivity group classified by the score of harm/care dimension showed the significant changes in recognition potentials between the moral and neutral conditions. In addition to the morality preference, prosocial behavior is an important form of reaction to moral events, which reflects the intention of the individual to help others (Eisenberg and Fabes, 1998; Malti et al., 2015; Turiel, 2015). Thus, specifically, in this study, we examined whether time perception of moral events relates to moral preference and prosocial tendency.

To this end, we designed two experiments using a temporal bisection task (Church and Deluty, 1977; Wearden, 1991; Nather et al., 2011; Fayolle et al., 2015; Jia et al., 2015). Participants first familiarized themselves with a short and a long standard duration, and then in the test they had to judge a given moral stimulus is close to the short or the long standard. Specifically, Experiment 1 conducted the temporal bisection task for the immoral and the neutral phrases, respectively. In addition, we measured the moral reference of individuals with the Moral Foundation Questionnaire (MFQ) (Graham et al., 2009, 2011) and tested their prosocial tendency with the prosocial tendency measure (PTM) (Carlo and Randall, 2002; Kou et al., 2007; Carlo et al., 2010). In Experiment 2, we introduced a further condition of disgust phrases, in addition to the immoral and neutral conditions, to distinguish the arousal account and embodiment account. According to the embodied time perception, the duration of immoral phrases can be lengthened relative to neutral and disgust phrases, if immoral phrases induce distinct embodied reactions. In addition, we expect that a strong moral preference and/or a strong prosocial tendency may enhance the difference of perceived duration between the immoral and neutral phrases. Alternatively, if immoral phrases only elicit negative emotion and elevate arousal similar to those disgust phrases, according to the arousal account, subjective durations of the immoral and disgust phrases should be comparable.

## Experiment 1

### Methods

#### Participants

A total of 26 university students (14 women; *M* = 20.96 years) from Jiangnan University were recruited for the experiment. The sample size was similar to the sample size (ranging from 18 to 25 participants) of previous studies on emotional modulation of time perception (Grondin et al., 2014; Droit-Volet and Berthon, 2017) and moral cognition (Yang et al., 2017). All the participants were native speakers of Chinese, with normal or corrected-to-normal vision. Before the experiment, each participant signed the form of informed consent. The study was approved by the Human Research Protections Program of Jiangnan University.

#### Stimuli and Apparatus

##### Word Phrases

Participants experimented individually in a quiet laboratory room, where they sat in front of a 24-inch LCD monitor (display resolution of 1,024 × 867 pixels and a refresh rate of 100 Hz) with a viewing distance of 57 cm. The target stimulus (subtended 3.9^°^ × 1.5^°^ visual angles) was a white phrase presented in the center of a black background. The left- and right-arrow keys on the keyboard were used as the response keys. The presentation of experimental stimuli and data records were generated through Matlab using the Psychophysics Toolbox (Brainard, 1997).

Two types of three-character Chinese phrases, with each of 20, were selected as the target stimuli: phrases depicting immoral behaviors (e.g., 杀老板, meaning “killing a boss”) and phrases indicating neutral behaviors (e.g., 擦桌子, meaning “wiping a table”).^1^ For each type of phrases, the first character (verb) denotes an action, and the last two characters combined as a noun subject. Each type of phrase and the corresponding descriptions are listed in Supplementary Table 1.

##### Measure of Moral Foundations Questionnaire

The 30-item Moral Foundations Questionnaire (MFQ-30) was to measure the moral preferences of individuals across five dimensions: harm/care, fairness/reciprocity, ingroup/loyalty, authority/respect, and purity/sanctity. This questionnaire includes two subscales: moral relevance and moral judgment. In the subscale of moral relevance, participants were required to judge the relevance for each of 15 items on a 6-point scale ranging from 0 (not at all relevant) to 5 (extremely relevant). For the subscale of moral judgment, participants had to rate their agreement with each of 15 items on a 6-point scale from 0 (strongly disagree) to 5 (strongly agree).

##### The Measure of Prosocial Tendencies

The PTM here was used to evaluate the prosocial behavior of university students. The PTM consists of six dimensions: public, anonymous, dire, emotional, compliant, and altruistic. Participants were asked to rate the extent to which the statements described themselves on a 5-point scale ranging from 1 (Does not describe me at all) to 5 (Describes me well). The average score for the 26 items was calculated, with a higher score indicating a higher level of prosocial behavior.

#### Procedure

Participants were first familiar with the short (400 ms) and long (800 ms) standard durations in a training session. A white rectangle (3.9^°^ × 1.5^°^ visual angles) was shown for either 400 or 800 ms, and participants had to identify the short (“S”) and the long (“L”) standard by pressing either the response keys labeled “short” or “long” on the keyboard. The training test consisted of 20 trials.

**FIGURE 1:**
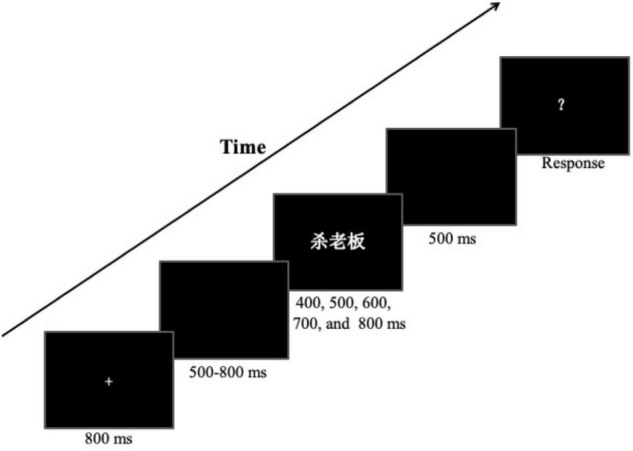
Schematic illustration of a trial procedure used in the experiment.

After participants got familiar with the short and long standards, a formal temporal bisection task started. Each trial started with a fixation cross (“+”) presented in the center of a screen for 800 ms, followed by a random 500 to 800 ms blank screen. Then a target phrase, randomly selected from the phrases list, was displayed for a given duration, randomly sampled from 400, 500, 600, 700, and 800 ms. After a 500-ms interval blank, a question mark appeared, prompting participants to judge whether the probe duration was closer to the “S” or the “L” by pressing the corresponding keys. The intertrial interval (ITI) was 2,000 ms. A schematic illustration of the stimulus presentation is shown in Figure 1. The formal temporal bisection task included 11 blocks with each of 20 trials. The first block was treated as a practice block and discarded in further analysis. Each experimental condition was repeated 20 times, and the different conditions were tested randomly trial by trial. In addition, at the beginning of each block, both the S and L standard durations were separately presented five times to remind participants of the short and long standards.

After the temporal bisection task, participants were asked to complete the two questionnaires (MFQ-30 and PTM) at their own pace.

### Results

#### Temporal Bisection

**FIGURE 2:**
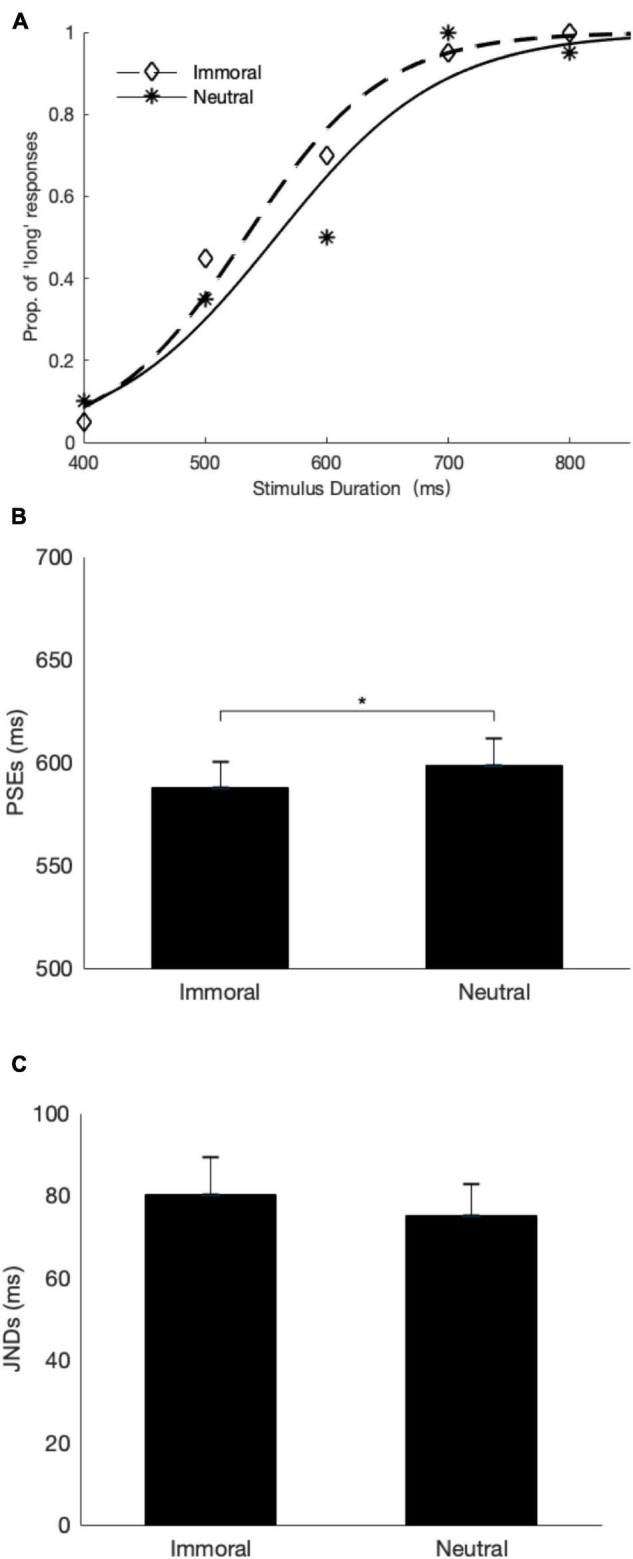
Temporal judgment results of Experiment 1. **(A)** Mean proportions of “long” responses (diamonds: the immoral phrase, stars: the neutral phrase) for a representative subject are plotted as a function of the probe durations, together with fitted psychometric curves (dashed: the immoral phrase, solid: the neutral phrase). **(B)** The mean PSEs and their respective one-standard errors for the two conditions. **(C)** The mean JNDs and their corresponding one-standard errors for the two conditions. *denotes *p* ≤ 0.05.

Figure 2A depicts the mean proportions of “long” responses as a function of the probe durations, separated for the immoral and neutral phrases with a fitted logistic psychometric curve for a representative participant. By visual inspection, the proportion of the “long” responses are generally higher for the immoral phrase relative to the neutral phrase. We then fitted individual psychometric curves and estimated the bisection point (BP) for each condition. The BP is the duration with which participants perceive neither closer to the short or the long [*p*(long) = 0.50, also known as the point of subjective equality, PSE]. The lower the BP is, the longer the duration is perceived. In addition, the just-noticeable difference (JND), an indicator of the temporal discrimination sensitivity, was estimated by calculating half of the difference limen between the 25 and 75% thresholds of the fitted curves.

The mean PSEs and JNDs for the immoral and neutral phrases are depicted in Figures 2B,C. A paired-sample *t*-test analysis revealed that the PSE was significantly lower for the immoral phrase (*M* = 588 ms) than for the neutral phrase (*M* = 599 ms), *t* (25) = –3.09, *p* < 0.01, Cohen’s *d* = 0.62, which indicates that the perceived duration of the immoral phrase was overestimated relative to the same duration of the neutral phrase. However, there was no significant JND difference in the temporal discrimination between two types of phrases, *t* (25) = 0.96, *p* = 0.35, Cohen’s *d* = 0.19, suggesting the discrimination sensitivity remained comparable across two conditions.

#### Relation Between the Scores on Moral Foundation Questionaire, Prosocial Tendency Measure, and the Point of Subjective Equality

**FIGURE 3:**
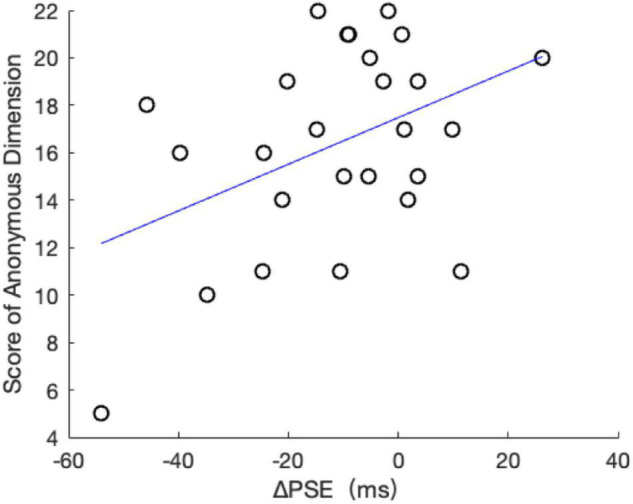
The scatter diagram between the ΔPSE and the score of anonymous dimensions from PTM.

To examine the correlation between the moral preference and/or prosocial tendency and the shift of subjective time, we first used the neutral condition as a baseline and measure the shift by the difference of PSEs (i.e., Δ*PSE = PSE_*immoral*_–PSE_*neutra*_*_*l*_). We then correlated this with the scores from MFQ^2^ and PTM. We failed to find any significant correlation between the MFQ score and the Δ*PSE*, *r* = 0.08, *p* = 0.70. The correlation between the total score of PTM and the Δ*PSE* was also nonsignificant, *r* = 0.14, *p* = 0.51. Interestingly, though, the score on the anonymous dimension of PTM was positively correlated with the shift of the PSE, *r* = 0.43, *p* = 0.03 (see Figure 3). In other words, the higher the anonymous prosocial tendency was, the larger the positive shift of PSE in the immoral phrase condition. Note, the positive shift in the PSE means that the overestimation of the immoral phrase is reduced. The anonymous score reflects the tendency that participants give anonymous help to others. In this study, its positive relationship with the ΔPSE might imply that participants who have a higher anonymous prosocial tendency pay more attention to the meaning of the immoral phrase (nontemporal processing) rather than the time processing of the stimulus, thus reducing the overestimation.

## Experiment 2

### Methods

#### Participants

A total of 24 university students from Jiangnan University 24 (12 females; *M* = 21 years) volunteered to take part in the experiment. All participants were native speakers of Chinese, with normal or corrected-to-normal vision. Each participant gave written informed consent before the experiment. The study was approved by the Human Research Protections Program of Jiangnan University.

#### Stimuli and Apparatus

The experimental design, stimuli presentation, and apparatus were the same as those in Experiment 1, except that Experiment 2 included a third type of phrase, describing disgusting behaviors (e.g., 吃鼻屎, meaning “eating booger”).^1^ In other words, the experiment involved three types of phrases: phrases depicting immoral behaviors, phrases describing disgusting behaviors, and phrases indicating neutral behaviors. The three types of phrases and the corresponding descriptions were listed in Supplementary Table 1.

#### Procedure

The structure of the procedure was the same as that used in Experiment 1, except for introducing the condition of disgusting-behavior phrases. Thus, the experiment consisted of 30 practice trials and ten blocks with each of 30 trials. The different types of phrases were tested randomly trial by trial.

### Results

**FIGURE 4:**
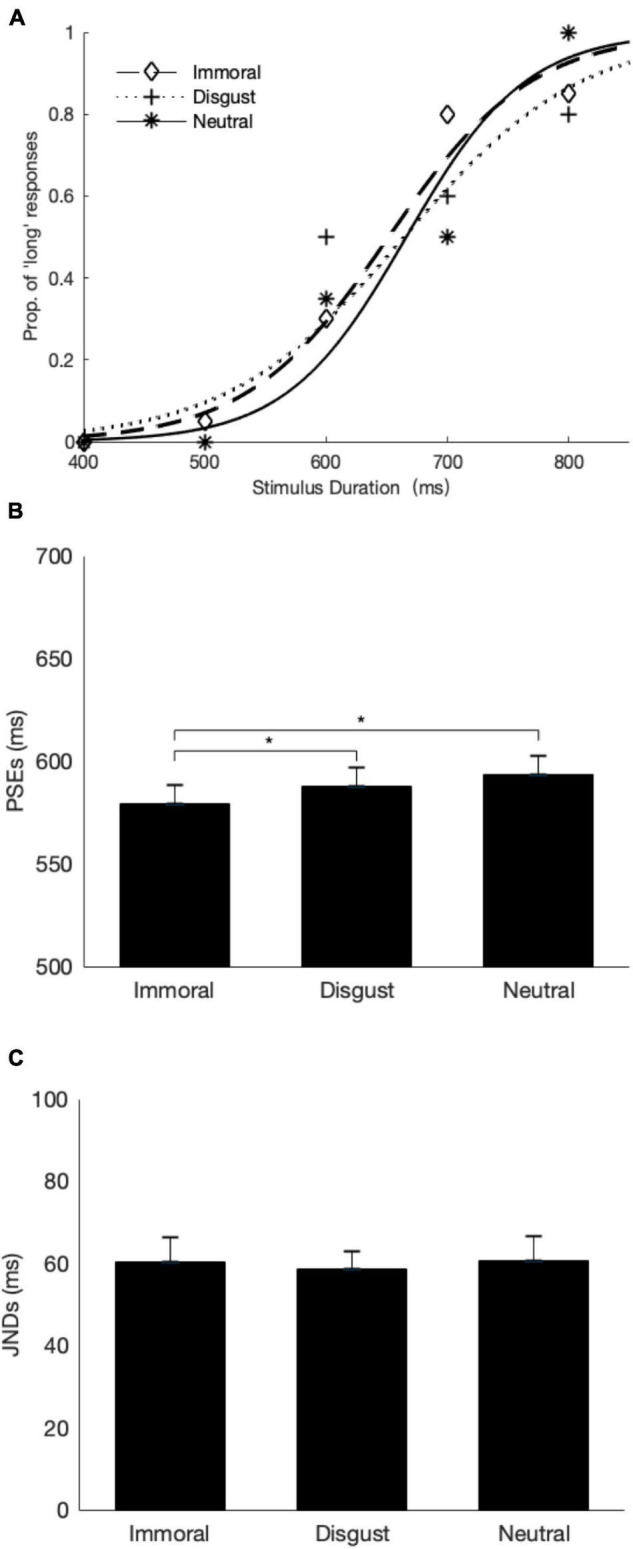
Temporal bisection results of Experiment 2. **(A)** The mean proportions of “long” responses from a representative subject are plotted (diamonds: the immoral phrase; crosses: the disgust phrase; and stars: the neutral phrase) against the probe durations, together with the fitted psychometric curves (dashed: the immoral phrase, dotted: the disgust phrase, and solid: the neutral phrase). **(B)** The mean PSEs and their respective one-standard errors are shown for the three conditions. **(C)** The mean JNDs and their respective one-standard errors are displayed for the three conditions. * denotes *p* ≤ 0.05.

Figure 4A shows the average psychometric curves for a representative participant. Figures 4B,C depict the mean PSEs and JNDs, respectively, for the three experimental conditions and all the participants. By visual inspection, the results were consistent with the findings of Experiment 1.

A repeated-measures ANOVA with the factor of phrase type revealed a significant main effect, *F* (2, 46) = 5.23, *p* < 0.01, η*_*p*_*^2^ = 0.19. A follow-up *post-hoc* comparison with Holm–Bonferroni correction revealed that the mean PSE was significantly lower for the immoral phrase (*M* = 579 ms) relative to the neutral phrase (*M* = 594 ms, corrected *p* = 0.01), and just reached significant level in comparison with the disgust phrase (*M* = 588 ms, corrected *p* = .05). Importantly, there was no significant difference between the disgust and the neutral phrases (corrected *p* = 0.27). Similar to Experiment 1, temporal discrimination sensitivities revealed in JNDs were comparable across three types of phrases, *F* (2, 46) = 0.24, *p* = 0.79, and η*_*p*_*^2^ = 0.01.

## Discussion

This study aimed to examine the influence of morality on time perception. We compared duration judgments of three types of phrases depicting immoral, disgust, and neutral behaviors. The results showed the immoral phrase was perceived longer in duration than the neutral and disgust phrases with the same physical duration, while the latter two did not differ from each other in duration judgments. In addition, we found a positive moderate correlation between the anonymous prosocial tendency and the difference in PSEs between the immoral and neutral phrases. The positive correlation indicates that the higher the anonymous prosocial tendency is, the less the overestimation would be for the immoral phrase.

The effect of social emotion on time perception has been mainly investigated with emotional facial expressions (Effron et al., 2006; Gil and Droit-Volet, 2011; Grondin et al., 2015). This study provides new evidence of social emotion on time perception with the moral-related stimuli. Immoral-related stimuli can also lengthen subjective time. One might argue that the finding is trivial, given that immoral stimuli are likely coupled with negative emotion and high arousal, which could be interpreted by the arousal account (Treisman et al., 1990; Penton-Voak et al., 1996). However, the arousal account could not fully explain the difference we observed between the immoral and disgust phrases, given that the disgust phrases had similar valence and arousal ratings as the immoral phrases. Rather, our findings can be better explained by discrete emotion theory (Izard and Ackerman, 2000; Mikels et al., 2005) and embodied time perception (Wittmann, 2014). According to discrete emotion theory (Izard and Ackerman, 2000), different types of behavioral functions link to different types of emotions, and vice versa. For example, both thread and disgust are categorized as high-arousal negative-valence emotions, but they activate different processes: threat activates our defense system, while disgust may merely activate avoidance. Similarly, seeing or doing different moral behaviors may link to different social reactions. Phrases depicting immoral behaviors (e.g., 踢老人, meaning “kicking an old person”) likely evoke people to blame this behavior or/and even be ready to rescue the victim. As a result, the intuitive social reaction lengthens the subjective time of the phrase. By contrast, phrases describing disgust behavior (e.g., 吃鸡屎, meaning “eating chicken shit”) may not invoke any social reaction, rather just feel the behavior is unacceptable.

Admittedly, previous studies have shown inconsistent results for the impact of disgust emotion on time perception. For example, the disgust-inducing pictures (e.g., mutilated body) were judged longer than the neutral and disgust faces (Grondin et al., 2014), whereas it has also been found no difference in duration to the neutral expressions (Gil and Droit-Volet, 2011) or even opposite, the presentation of disgust food shortened its subjective duration (Gil et al., 2009). Droit-Volet et al. (2013) have suggested that time perception of a disgust stimulus depends on whether the stimulus is perceived as relevant or not. Participants seem to regard the observation of disgusting expression of another person as irrelevant, whereas the disgusting scenes probably prompt participants to react as quickly as to avoid possible harm. Disgust food, however, might activate participants to protect their health, which requires more attention toward this kind of food and thus less attention is given to temporal processing. In short, specific meanings of disgust stimuli and the correspondent reactions determine the perceived time. Compared with disgusting scenes and disgust food, semantic level of disgust expression, such as disgust phrases we used in this study, may not be strong enough to induce implicit reaction (As shown in Figure 4B, there was a numerical reduction, but not significant, for the disgusting phrase relative to the baseline neutral phrase).

Another interesting finding of this study is that the shift of PSE of the immoral phrase relative to the neutral phrase was correlated with the score of the anonymous dimension of PTM. The anonymous dimension is defined as the willingness to help without others’ knowing (Carlo et al., 2010). When an observer has a higher relative to a lower level of anonymously prosocial trait, they are likely captured more by the nontemporal semantic processing of the immoral phrase (i.e., interpretation of its meaning and consequence) relative to the temporal processing (i.e., monitoring the passage of time) (Zakay and Block, 1996). As a consequence, those observers with high anonymously prosocial traits had less overestimation with the immoral phrase as compared to those with the low anonymously prosocial trait. Interestingly, though, the overestimation did not correlate with the moral reference. The possible reason is that the moral reference mainly relates to the later decision-related processing (Palmer et al., 2013; Zhang et al., 2016), but might not have a direct linkage with the moral-induced temporary internal states (e.g., high arousing, potential embodiment), the latter determining timing process.

Recall the review in the introduction, the duration lengthening effect in recent studies is usually attributed to the arousal or/and the attention mechanism under the framework of the pacemaker-accumulator clock (Gibbon and Church, 1990), and the implicit reaction mediated by the relevance to our body (Wittmann and van Wassenhove, 2009; Droit-Volet et al., 2013; Wittmann, 2014). Our findings distinguish the role of implicit reaction induced by moral-related information from that of arousal in perceived duration. Both the immoral- and disgust-behavioral phrases were categorized as the negative and high-arousal stimuli, but only the immoral-behavioral words lengthened the subjective duration relative to the neural-behavioral words. The absence of duration distortion for disgust-behavioral words further supports that the embodied reaction is a key factor influencing duration perception (Gil and Droit-Volet, 2011; Shi et al., 2012; Jia et al., 2015; Droit-Volet, 2019).

Still, one might wonder whether the number of experimental conditions would affect the duration judgment as contextual bias. It has been shown that the experimental context could affect the temporal judgment by mediating participants’ prior knowledge and prediction of the forthcoming stimulus (Shi et al., 2013; Ulrich and Bausenhart, 2019; Glasauer and Shi, 2021a,b; Zhu et al., 2021). For example, the frequency of the probe durations can cause a shift of the bisection point toward the ensemble mean of the sample durations (Zhu et al., 2021). However, this is unlikely here given that all conditions were tested in the same range with a same number of trials. Introducing the third category disgust phrases in Experiment 2, however, might increase the variability of the test stimuli as compared to Experiment 1. This might introduce more working memory resources to represent different categories in Experiment 2 relative to Experiment 1, which could potentially cause a contrast effect in working memory. In other words, observers might bias temporal judgments of different categories into separate temporal ranges (e.g., one was biased toward the short and the other toward the long) to maintain the discriminability among the categories. However, this is also unlikely given that the difference between the PSEs of the two extremes (the immoral and neutral phrases) was comparable in Experiment 1 and 2 (11 and 15 ms, respectively).

In summary, this study showed that the immoral phrase lengthens its subjective duration, while the disgust-behavioral words did not. The findings favor the important role of moral-induced intuitive reaction on temporal processing, which is distinct from the arousal-based lengthening effect.

## Data Availability Statement

The raw data supporting the conclusions of this article will be made available by the authors, without undue reservation.

## Ethics Statement

The studies involving human participants were reviewed and approved by the Human Research Protections Program of Jiangnan University. The patients/participants provided their written informed consent to participate in this study.

## Author Contributions

LJ, ZS, and XW developed the design of the study and contributed to writing and drafting the manuscript. BS collected data. LJ, BS, and ZS performed the data analyses. All authors contributed to the article and approved the submitted version.

## Conflict of Interest

The authors declare that the research was conducted in the absence of any commercial or financial relationships that could be construed as a potential conflict of interest.

## Publisher’s Note

All claims expressed in this article are solely those of the authors and do not necessarily represent those of their affiliated organizations, or those of the publisher, the editors and the reviewers. Any product that may be evaluated in this article, or claim that may be made by its manufacturer, is not guaranteed or endorsed by the publisher.

## Footnotes

1 The three types of phrases in the experiment were adopted and modified from the phrases used by Yang et al. (2017). A total of 90 words, with each type of 30, were tested before the experiment by a separated group of participants (20 in total, 11 women, *M* = 19.25), who judged the subjective feeling of the depicted behaviors: immoral, disgust, or neutral. Phrases were selected for the formal experiment when the phrase was classified correctly above 75%. In addition, participants were asked to rate the arousal (from very calm to very exciting) and valence (from very unpleasant to very pleasant) for each phrase on a 9-point Likert scale of self-assessment-manikin (SAM) (Bradley and Lang, 1994). To ensure that both immoral- and disgust-behavior phrases had the same level of negative and high-arousal, we only selected those phrases that were rated as higher than 5 in arousal and lower than 5 in valence. In addition, we selected those neutral phrases that were evaluated in arousal lower than 5 and in valence between 4 and 6. Based on these criteria, 20 immoral, 20 disgust, and 20 neutral behavior phrases were selected as formal experimental stimuli (Supplementary Table 1). Both the immoral- and disgust-behavior phrases were evaluated as more arousing and negative than the neutral phrases (all *ps* < 0.001), while the immoral and disgust phrases were comparable in emotional arousal and valence (*ps* > 0.1). For the number of strokes, both the immoral- and the disgust-behavior phrases were matched with the neutral-behavioral phrases (*ps* > 0.1).

2 One participant was excluded for this correlation analysis, because the score of MFQ was beyond 2.5 SDs of the average score (*M* = 91.82, *SD* = 18.14).
