# Supplementary material for: Phrase Depicting Immoral Behavior Dilates Its Subjective Time Judgment
Source: Front Psychol. 2021 Dec 24;12:784752. doi: 10.3389/fpsyg.2021.784752 (PMC8739786; doi:10.3389/fpsyg.2021.784752)
Supplement: Supplementary file 1 [file Table_1.pdf]

APPENDIX

Table 1 | Phrases used in the present study

Type	Phrase	Valence	Arousal
Immoral	养情人	2.55	6.60
	卖假药	2.05	5.55
	打警察	2.25	5.75
	骂老师	2.95	5.70
	卖假肉	2.65	5.10
	骗钱财	2.10	5.90
	打医生	1.85	6.40
	拐小孩	1.90	6.20
	偷手机	2.10	6.15
	打学生	1.85	6.25
	卖毒米	1.80	6.05
	踢小孩	2.25	6.25
	打护士	2.20	6.10
	杀老板	2.50	5.80
	骂长辈	1.95	6.30
	踢路人	2.60	5.20
	打老婆	1.25	7.90
	踢老人	2.05	6.45
	踩小猫	1.50	6.65
	杀男友	2.25	6.35
Disgust	吃蛔虫	1.55	6.40
	玩粪便	1.80	6.55
	吃鸡屎	1.90	5.65
	玩狗屎	1.90	6.10
	摸蛔虫	2.00	6.30
	踢大便	1.80	6.20
	吃鼻屎	1.90	5.75
	吃腐肉	2.00	5.25
	抹浓痰	1.70	6.35
	捏蛆虫	1.85	6.60
	吃粪便	1.55	7.10
	舔鼻涕	2.40	5.45
	吃黏虫	1.65	6.15
	喝脓血	1.90	6.70
	戳大便	1.80	6.40
	踢狗屎	2.20	5.30
	闻鸡屎	2.00	6.05
	舔鼻屎	2.20	5.30
	闻羊屎	2.25	5.55
	踩牛粪	2.65	5.40
Neutral	擦桌子	4.80	3.20
	点鼠标	5.00	3.00
	卖衣服	5.30	3.75
	拍皮球	5.50	3.70
	穿衣服	5.55	3.50
	梳头发	5.55	4.20
	戴眼镜	4.25	3.10
	拖地板	4.75	3.20
	洗盘子	4.30	3.35
	修电脑	4.55	4.35
	编辫子	5.55	4.05
	搬砖头	4.55	3.40
	叠被子	4.70	2.75
	乘动车	5.10	3.90
	骑单车	5.55	3.70
	搬箱子	4.40	3.15
	等公交	4.45	3.10
	配钥匙	4.80	3.50
	削铅笔	4.85	2.90
	看手表	5.20	3.80
